# Antimicrobial effect of Licochalcone A and Epigallocatechin-3-gallate against *Salmonella* Typhimurium isolated from poultry flocks

**Published:** 2018-02

**Authors:** Somayyeh Hosseinzadeh, Habib Dastmalchi Saei, Malahat Ahmadi, Taghi Zahraei Salehi

**Affiliations:** 1 Department of Microbiology, Faculty of Veterinary Medicine, Urmia University, Urmia, Iran; 2 Department of Microbiology, Faculty of Veterinary Medicine, Tehran University, Tehran, Iran

**Keywords:** Licochalcone A, Epigallocatechin-3-gallate, Drug resistance, Biofilm, *Salmonella* Typhimurium

## Abstract

**Background and Objectives:**

Salmonellosis due to multi-drug resistant *Salmonella* Typhimurium with biofilm formation ability is a serious public health threat worldwide. Studies have shown that medicinal plants inhibit the growth of bacterial species. The present study aimed at determining antibiotic resistance pattern and biofilm formation ability of *S.* Typhimurium isolated from poultry flocks. Moreover, the antibacterial activity of Licochalcone A (LAA) and Epigallocatechin-3-gallate (EGCG) against the studied isolates were investigated in this study.

**Materials and Methods:**

Antibiotic susceptibility testing of *S*. Typhimurium RITCC1730 and 23 clinical isolates of *S.* Typhimurium against 8 antibiotics was performed using standard Kirby-Bauer disc diffusion method. The extent of biofilm formation was measured by Microtiter dish biofilm formation assay. Antimicrobials activities of LAA and EGCG were determined by MIC and MBC assays using microdilution method.

**Results:**

The highest antimicrobial resistance was detected against chloramphenicol (52.17%), followed by furazolidone (26.08%), and trimethoprim/sulfamethoxazole (21.73%). All isolates were sensitive to ciprofloxacin (100%), followed by gentamicin, imipenem (95.65%), and cefixime (91.30%). Most of the isolates (78.26%) were able to produce weak biofilm. LAA and EGCG inhibited the growth of *S.* Typhimurium at the MIC levels of 62.5∼1000 and 1.56∼400 μg/mL, respectively. The MBC value of LAA was >1000 μg/mL, while the corresponding value of EGCG varied from 100 to 800 μg/mL.

**Conclusion:**

*S.* Typhimurium isolates revealed a multiple antibiotic resistance with biofilm production ability. As a result, EGCG, and to a lesser extent, LAA displayed potential antibacterial activity against *S*. Typhimurium and could be considered as useful compounds for the development of antibacterial agents against salmonellosis.

## INTRODUCTION

Members of the *Salmonella* genus are enteric bacteria, and salmonellosis is a worldwide disease that infects both humans and animals. *Salmonella enterica* subsp. *enterica* serovar Typhimurium (*Salmonella* Typhimurium) is the most common serovar, which is widely distributed as a food-borne pathogen, and it is one of the most prevalent causes of bacterial food-borne diseases in humans and animals (1).

Many serovars of *Salmonella* have been reported from poultry worldwide, of which *S.* Typhimurium is of prime importance. Salmonellosis in poultry is an important area of study as it not only affects the poultry industry, but it can also transmit to humans through the consumption of contaminated poultry products (1, 2).

In recent years, the wide use of antibiotics in the diet of domestic animals has become a threat for the world’s population as it increases the occurrence of bacterial resistance against available antibiotics (3). Also, antimicrobial drug resistance among *Salmonella* serovars has increasingly been reported in Iran and other countries (2, 4, 5); among them, *Salmonella enterica* serovar Typhimurium has been reported to show multi-drug resistance (6, 7). The problem of antibiotic resistance of *Salmonella* becomes more important when the biofilm formation ability of these bacteria is considered. Biofilm was defined as “dominant lifestyle of microorganisms attached to a biotic or abiotic surface and embedded within self-produced extracellular polymeric substances” (8). Biofilm formation ability is reported to be widespread among isolates of *Salmonella* spp. (9). It was shown that biofilm formation can lead to increased resistance to drugs and to chemical, physical, and mechanical stresses and may interfere with the host immune system (10).

Therefore, treatment of salmonellosis using medicinal plants has become essential, especially with the new trend of antibiotic resistance. Licochalcone A (LAA), a natural plant product, is a retrochalcone purified from the roots and rhizomes of *Glycyrrizainfata* (11). Epigallocatechin-3-gallate (EGCG) is a major polyphenol found with high concentrations in the leaves of *Camellia sinensis* (green tea). Both of these compounds have various biological activities, eg, anti-oxidative, anti-protozoal, anti-mutagenic, anti-inflammatory, and cancer-preventive properties, anti-biofilm formation, and anti-quorum sensing (11, 12).

Many researches have reported that LAA and EGCG have antibacterial activity against both Gram-positive and Gram-negative bacteria (11–14). Although the antimicrobial effects of LAA and EGCG have previously been investigated against some bacterial species, there is scarcity of data on the effects of these compounds on *S*. Typhimurium isolated from poultry with salmonellosis. Thus, such an investigation may introduce LAA and EGCG as new therapeutics for the treatment of salmonellosis in poultry. Therefore, the objectives of this study were to determine the antibiotic resistance pattern and ability of biofilm formation of *S.* Typhimurium isolated from poultry flocks and to investigate the antimicrobial effect of LAA and EGCG against these isolates using broth microdilution method.

## MATERIALS AND METHODS

### Bacterial strains and materials.

A total of 23 clinical isolates of *S*. Typhimurium, which were isolated from poultry flocks, were kindly provided by Dr. T. Zahraei-Salehi of Faculty of Veterinary Medicine, Tehran, Iran. These isolates had previously been identified by biochemical and molecular tests to be representative of *S*. Typhimurium (15). *S.* Typhimurium RITCC1730 was also obtained from Razi Institute Culture Collection Center. LAA and EGCG were purchased from Sigma-Aldrich (Germany), and a stock solution was made in dimethyl sulfoxide (DMSO; Sigma-Aldrich). The final concentration of DMSO for dissolving compounds was 10% (v/v). The LAA and EGCG stock solutions concentration were 2 mg/mL and 0.8 mg/mL, respectively.

### Antibiotic sensitivity tests (antibiogram test).

All *S.* Typhimurium isolates were subjected to in vitro antibiotic susceptibility testing against 8 antibiotics of different classes. Disk diffusion method was used following the guidelines of Clinical and Laboratory Standards Institute (16). Antibiotics used in the study were cefixime (5 μg), chloramphenicol (30 μg), ciprofloxacin (5 μg), enrofloxacin (5 μg), furazolidone (100 μg), gentamicin (10 μg), imipenem (30 μg), and trimethoprim/sulfamethoxazole (1.25/23.75 μg). All antibiotic disks were procured from PadtanTeb Company and Roshdlab (Iran). *Salmonella* isolates that demonstrated resistance to 2 or more antibiotics were considered as multi-drug resistance strains (17).

### Biofilm formation.

The procedure of biofilm formation of isolates in polystyrene microtiter plates was based on the previously described method with some modifications (18). Briefly, 200 μL of bacterial suspension with OD600 = 0.1 (10^7^ log CFU/ml) was inoculated directly to each well using 3 wells per isolate. Plates were wrapped with parafilm and incubated at 37°C for 24 hours. Then, the plates were washed 3 times by phosphate buffered saline (PBS) (Sigma-Aldrich) and allowed to air-dry for 20 minutes. In the next step, biofilms were stained with 150 μL of 1% w/v crystal violet (CV) for 30 minutes, washed twice with taped water to remove excess stain and were then allowed to air-dry for 30 minutes. Biofilm was quantified by eluting CV with 150 μL 95% v/v ethanol and determining the optical absorbance of the eluted dye at 570 nm. Ethanol (95%) was used as blank control. The optical density cut-off (ODc) was defined as the mean OD of the negative control (culture medium), and the isolates were classified as follow: non-adherent (OD ≤ OD_c_); weak adherent (OD_c_< OD ≤ 2xOD_c_); moderate adherent (2x OD_c_< OD ≤ 4 x OD_c_); and strong adherent (OD > 4xOD_c_) (18). Microscopic examination of the wells was directly performed under oil immersion with transmitted light using an Olympus CH40 system microscope.

### Determining minimum inhibitory concentration (MIC).

The minimum inhibition concentrations (MICs) of LAA and EGCG against *S.* Typhimurium RITCC1730 and 23 other isolates were determined in triplicate by broth microdilution method using twofold serial dilutions in MHB, according to the standard CLSI procedures (16). Each plate had a set of controls: positive, growth, sterility, and solvent. A column with a ciprofloxacin antibiotic in serial dilution was used as a positive control, a column with all solutions except for the test compounds as growth control, a column as sterility control (test compound in serial dilution + broth + indicator), and a column as solvent control (solvent in serial dilution + broth + indicator). Ciprofloxacin (1 mg/mL) was included as positive control, *S.* Typhimurium RITCC1730 as growth control, LAA (2 mg/mL) and EGCG (0.8 mg/ mL) as sterility control, and DMSO as solvent control. After incubation for 24 hours at 37°C, resazurin (0.01 %) was added to all wells (30 μL per well) and was further incubated for 2 to 4 hours to observe color change. Color change from blue to pink indicates reduction of resazurin, and therefore, bacterial growth. After incubation, the lowest concentration of LAA or EGCG, at which color change occurred, was recorded as the MIC value.

### Determining minimum bactericidal concentration (MBC).

The minimum bactericidal concentration (MBC) was recorded as the lowest concentration of LAA or EGCG, which killed 99.9% of bacterial inoculate after a 24- hour incubation at 37°C (19). MBC values were determined by removing 100 μL of bacterial suspension from subculture, demonstrating blue color in wells and inoculating on Muller Hinton agar plates. Plates were incubated at 37°C for a total period of 24 hours. Each experiment was performed in triplicate.

## RESULTS

### Antibiotic sensitivity tests (antibiogram test).

Among the studied *S.* Typhimurium isolates, 12 (52.17%), 6 (26.08%), 5 (21.73%), and 1 (4.34%) were resistant to chloramphenicol, furazolidone, trimethoprim/sulfamethoxazole, and enrofloxacin, respectively. All isolates of *S.* Typhimurium were susceptible to ciprofloxacin, gentamicin, imipenem, cefixime, enrofloxacin, trimethoprim/sulfamethoxazole, chloramphenicol, and furazolidone (Table 1). *S*. Typhimurium RITCC1730 was sensitive to all antibiotics although sensitivity against furazolidone was intermediate.

**Table 1.** Antibiogram results of the studied *Salmonella* Typhimurium isolates

**Antibiotics**	**Symbol**	**Concentration (μg/disc)**	**Class**	**Sensitive n (%)**	**Intermediate n (%)**	**Resistant n (%)**
Cefixime	CFM	5	Cephalosporin	21 (91.30)	2 (8.69)	0 (00.00)
Chloramphenicol	C	30	Miscellaneous	11 (47.82)	0 (0.00)	12 (52.17)
Ciprofloxacin	CP	5	Quinolone	23 (100.00)	0 (0.00)	0 (0.00)
Enrofloxacin	NFX	5	Quinolone	19 (82.60)	3 (13.04)	1 (4.34)
Furazolidone	FX	100	Nitrofuran	3 (13.04)	14 (60.86)	6 (26.08)
Gentamicin	GM	10	Aminoglycoside	22 (95.65)	1 (4.34)	0 (0.00)
Imipenem	IPM	30	Carbapenem	22 (95.65)	1 (4.34)	0 (0.00)
Trimethoprim/sulfamethoxazole	SXT	1.25/23.75	Potentiated Sulphonamides	18 (78.26)	0 (0.00)	5 (21.73)

Among the 23 isolates of *S.* Typhimurium, 7 (30.43%) were resistance to 1 antibiotic and 7 (30.43%) were regarded as multi-drug resistant (MDR) species. The remaining isolates (39.13%) were found to be sensitive or intermediate against the studied antibiotics (Table 2).

**Table 2.** MICs and MBCs value concentration of LAA and EGCG, biofilm formation ability and antibiotic resistance pattern of the studied isolates

**Sample N.**	**MIC LAA (μg/ml)**	**MBC LAA**	**MIC EGCG (μg/ml)**	**MBC EGCG**	**Biofilm formation ability**	**Antibiotic resistance pattern**
B32	1000	>1000	200	800	+	NFX/FX/C^a^
B33	250	>1000	6.25	400	+	-
B35	250	>1000	3.125	100	+	-
E20	250	>1000	6.25	400	−	-
E21	1000	>1000	400	800	+	FX/C^a^
E22	125	>1000	6.25	800	+	-
E23	250	>1000	200	400	+	C
E24	250	>1000	200	400	+	C
E25	1000	>1000	400	800	+	FX
E26	125	>1000	200	400	+	C
E27	250	>1000	50	200	+	C
E28	1000	>1000	200	800	+	FX/SXT^a^
D21	250	>1000	400	800	−	-
D22	1000	>1000	400	800	−	FX/SXT/C^a^
D23	1000	>1000	200	800	+	SXT/C^a^
D24	125	>1000	200	400	+	C
D25	125	>1000	200	800	−	C
D26	125	>1000	400	800	+	SXT/C^a^
D27	1000	>1000	400	800	+	FX/SXT/C^a^
H22	1000	>1000	1.562	100	+	-
H23	62.5	>1000	200	800	−	-
H24	125	>1000	6.25	400	+	-
H26	125	>1000	400	800	+	-
*Sal.*	125	>1000	6.25	400	−	-
RITCC1730						

a Multi-drug resistant isolates; Chloramphenicol (C); Enrofloxacin (NFX); Trimethoprim/sulfamethoxazole (SXT); Furazolidone (FX)

### Biofilm formation.

**Fig. 1.**
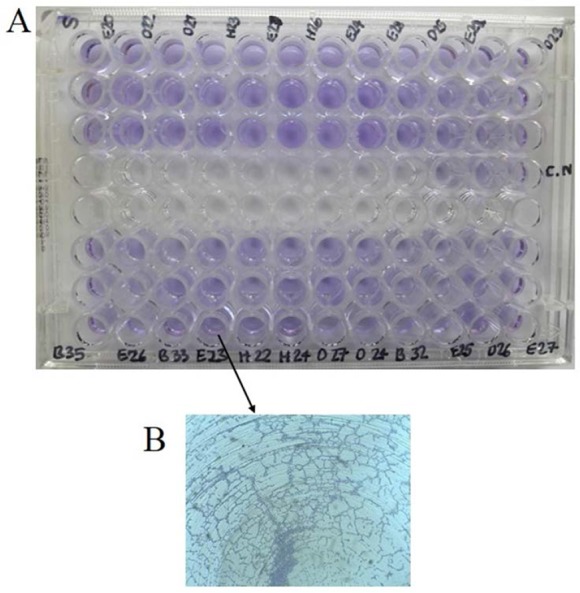
A; Weak biofilm type of *S.* Typhimurium isolates on polystyrene microtiter, B; Micrograph of biofilm producing isolates.

None of the isolates could produce a strong biofilm. Among the 23 *S*. Typhimurium isolates, 78.26% (18/23) showed to have weak adherence to polystyrene microtiter plates and were considered as presumptive biofilm producers. The remaining isolates and *S*. Typhimurium RITCC1730 were unable to produce biofilm (Table 2). Micrograph of weak biofilms formation of isolates are presented in Fig. 1.

### Determining minimum inhibitory concentration (MIC).

The MICs of LAA and EGCG against 23 *S*. Typhimurium isolates ranged from 62.5–1000 μg mL^−1^ and 1.56–400 μg mL^−1^, respectively. Also, the MIC value of LAA and EGCG against *S*. Typhimurium RITCC1730 was 125 μg mL^−1^ and 6.25 μg mL^−1^, respectively. MICs value of LAA and EGCG against 23 *S.* Typhimurium isolates and *S*. Typhimurium RITCC1730 are presented in Table 2.

### Determining minimum bactericidal concentration (MBC).

The MBCs value of LAA against all isolates was >1000 μg mL^−1^ while the corresponding value of EGCG varied against isolates and ranged from 100 to 800 μg mL^−1^. The MBC value of EGCG against *S*. Typhimurium RITCC1730 was 400 μg mL^−1^ (Table 2).

## DISCUSSION

The widespread emergence of resistance to antimicrobial agents in pathogenic bacteria from animal origins has become a significant global threat for public health. Chloramphenicol is one of the antibiotics that has been widely used in veterinary practice because of its broad spectrum antimicrobial activity (20). Results of the present study revealed that high levels of drug resistance to chloramphenicol were found in *S*. Typhimurium isolates (52.17%). This finding could be supported by recent studies from Iran and other regions of the world. In the survey of Ghoddusi et al. in 2015 (21) and Fallah et al. in 2013 (22), resistance to chloramphenicol in *Salmonella* spp. isolates from chickens was reported to be 70% and 64%, respectively. Also, resistance to chloramphenicol has been observed in another serovars of *Salmonella enterica* in animals worldwide (23, 24). On the other hand, some of the studies have reported *Salmonella* serovars resistance to conventional antibiotics such as furazolidone, enrofloxacin, trimethoprim/sulfamethoxazole, and other newer antibiotics with increasing frequency in many areas of the world (4, 25, 26). In this study, resistance to furazolidone, trimethoprim-sulfamethoxazole, and enrofloxacin was observed with low percentages (Table 1). In total, the reasons behind the resistance of isolates are the uncontrolled use of antibiotics in veterinary medicine that cause destruction of sensitive bacteria and selection of resistance species to multiple antibiotics.

Based on the studies, resistance to 2 or more antibiotics is defined as a multi-drug resistance (17). Our results indicate that among the *S*. Typhimurium isolates, 30.43% showed resistance to 2 or 3 antibiotics. The most antibiotic resistance patterns were FX/SXT/C (2; 8.69%) and SXT/C (2; 8.69%). These results are in agreement with those of Stevenson et al. in 2007 (5) and Fallah et al. in 2013 (22), which reported that the common resistance pattern was towards chloramphenicol and trimethoprim/sulphamethoxazole. In this study, *S*. Typhimurium isolates were sensitive to ciprofloxacin (100%), gentamicin (95.83), imipenem (95.83%), and cefixime (91.66). These results are similar to those reported by other studies, which were performed from 2004 to 2013 (2, 22, 27).

Biofilms formation by bacteria are directly associated with many infections. Different studies were conducted to compare the ability of different *Salmonella* serovars with respect to biofilm production (28, 29). Most of the *S*. Typhimurium isolates (78.26%) in our study possessed the ability to produce biofilm on polystyrene microtiter plate, however, all these isolates were weak biofilm producers. This result is in accordance with that of recent studies indicating that most of the *Salmonella* isolates were weak biofilm producers (30, 31). The differences in biofilm formation could be attributed to strain variation (29). It is interesting to note that from 18 isolates capable of biofilm formation, only 6 were multi-drug resistant. Thus, it can be suggested that biofilm formation acts as a mechanism for bacteria to survive better, especially in isolates with insufficient resistance level (32).

Medical plants have shown variety of antimicrobial activities and they have been found to cure many infections (33). The findings of our study showed that LAA has a good inhibitory effect on *S*. Typhimurium with a minimum inhibitory concentration in the range of 62.5 to 1000 μg mL^−1^ and minimum bactericidal concentration of >1000 μg mL^−1^. These results are in agreement with those of Tsukiyama (13) and Hantano (14) who showed LAA was effective in Gram- negative bacteria such as *Escherichia coli* and *P. aeruginosa*, with a similar range. Also, our results demonstrated that EGCG has inhibitory effects on *S*. Typhimurium isolates, with the MICs approximately in the range of 200 to 400 μg mL^−1^. MBCs values of this compound ranged from 100 to 800 μg mL^−1^. These results are consistent with data obtained in some studies that showed EGCG exerts antibacterial effects against food-borne pathogenic Gram- negative bacteria including *Helicobacter pylori*, enterohaemorrhagic *E. coli* (EHEC), *Vibrio cholera, Shigella* spp. and *Salmonella* spp. (12).

In our study, MICs and MBCs values of LAA against isolates were higher than those of EGCG. Thus, these results showed that EGCG was more effective than LAA. This result may be explained by the fact that antimicrobial mechanisms of these 2 compounds are different. Antimicrobial agents are often categorized according to their principal mechanism of action (34).

Several reports have shown that the minimum inhibitory concentration of LAA and EGCG against Gram- positive bacteria was lower than Gram- negative bacteria (11, 12). Although LAA had been proved to have activity against bacteria, its antimicrobial mechanism was not well- elucidated. One possible reason for the high concentration of LAA for Gram-negative bacteria could be the weak penetration of the LAA through the cell wall of the bacteria (35). One mode of EGCG action is binding to peptidoglycan (12). Peptidoglycan in Gram- negative bacteria is overlaid by an outer membrane, which is mainly composed of lipopolysaccharide. For this reason, it was hypothesized that physiological function of this layer and low affinity between EGCG and LPS limited the binding of EGCG to peptidoglycan in low concentrations. However, conducting further studies with more focus on antimicrobial mechanisms of LAA and EGCG is suggested.

## CONCLUSIONS

The results of this study revealed that the occurrence of MDR in *S*. Typhimurium isolates originated from poultry flocks with capability of weak biofilm production. The research has also shown that LAA and EGCG inhibit the growth of *S.* Typhimurium and have the potential to be developed in to a new class of antibiotics. However, conducting extensive studies with a large number of isolates and further in vivo analysis are needed to validate the antimicrobial properties of LAA and EGCG against *S*. Typhimurium.
